# Mapping Positions on Forgiving an Aggressor in Sport

**DOI:** 10.3389/fpsyg.2021.561031

**Published:** 2021-02-19

**Authors:** Eric Fruchart, Patricia Rulence-Paques

**Affiliations:** ^1^Laboratoire Interdisciplinaire Performance Santé Environnement de Montagne, UR 4604 Université de Perpignan Via Domitia, UFR STAPS, Font-Romeu-Odeillo-Via, France; ^2^EA4072 Laboratoire PSITEC, Université Charles-de-Gaulle Lille 3, Villeneuve-d’Ascq, France

**Keywords:** mapping, forgiveness, aggression, sport, information integration

## Abstract

The objective of the present study was to map amateur athletes’ positions on forgiving an aggressor in sport under various circumstances. One hundred and twenty-eight participants judged forgiveness in 32 scenarios built from combinations of five factors (moral disengagement, intention, consequence, apology, and incentive). Following a cluster analysis, ANOVAs, and chi-squared tests, a three-cluster solution was found: “Mainly Forgive, with Non-Additive Integration,” “Seldom Forgive, with Additive Integration,” and “Moderately Forgive, with Additive Integration.” The clusters’ composition was related to the members’ sex and type of sport. Cluster 1 contained 19% of the women and 32% of the athletes from collision sports. Cluster 2 contained 72% of the men, 53% of the athletes from non-contact sports, and 43% of the athletes from contact sports. Cluster 3 contained 54% of the women, and 58% of the athletes from collision sports.

Inter-individual relationships are common in contact team sports, and participants may be subjected to various types of aggressive behavior by their opponents (e.g., Tenenbaum et al., 1997). Some aggressive behaviors are hostile, i.e., with the intention of injuring the opponent. Others are instrumental, i.e., with the intention of attaining a certain objective within the game. Whatever the degree of contact in the sport and the type of aggressive behavior, a participant may sometimes forgive his/her attacker (Bar-Eli et al., 2016). The issue of forgiveness in sport is important because it has a positive effect on social relationships (Anderson, 2008).

Psychologists have used information integration theory to study forgiveness in various domains (for a review, see Anderson, 2008). For instance, Gauché and Mullet (2005) highlighted differences in the influence of apologies, intent to harm, cancelation of consequences, social proximity, and attitude of others on willingness to forgive in two cases: physical aggression and psychological aggression in the workplace, and physical aggression in sport. These differences did not depend on the participant’s sex or age. Gauché and Mullet (2005) suggested that other modalities of factors or other factors should be studied in order to better understand willingness to forgive in a sports context, using information integration theory.

Information integration theory (Anderson, 2008) allows one to identify the manner in which people cognitively integrate various factors when making a decision or judging a situation. The theory highlights the cognitive algebra operations people use to process information in various situations. Cognitive algebra involves additive or non-additive rules (for an illustration in sport, see Rulence-Pâques et al., 2005). With an additive rule, factors are given the same weight and a graphical analysis shows parallel curves. With a non-additive rule, factors are not given the same weight and the curves are not parallel. This theoretical framework has also been used to characterize judgment positions in various domains. In the sporting domain, various moral judgments (Fruchart and Rulence-Pâques, 2014; Fruchart et al., 2019) and judgments of well-being (Fruchart and Rulence-Pâques, 2020) have been described. These judgments were related to the person’s characteristics, such as age or the level of involvement in sport. In a study of the political domain in Colombia, López-López et al. (2012) mapped positions on forgiving individuals who had been more or less actively involved in the violence that has ravaged the country over the past 60 years. These results highlighted the value of information integration theory (Anderson, 2008) in mapping the way people combine various factors when judging an act in sport or when judging willingness to forgive.

A variety of factors influence whether individuals would forgive an aggressive act un sport (Gauché and Mullet, 2005). Firstly, the aggressor often apologizes to the victim, and the two may shake hands warmly at the end of the match. Aggression is an integral part of contact sports, and apologizing for an intentionally aggressive act is part of the “sporting spirit” (Kavussanu, 2012). Secondly, intention can be studied by looking at the dichotomy between the intention to inflict harm, (i.e., hostile aggression) and the intention to attain a desired objective (i.e., instrumental aggression) (e.g., Bar-Eli et al., 2016). Athletes are capable of rapidly analyzing a foul and determining whether or not it was intentional (Bar-Eli et al., 2016). Thirdly, through the mechanisms of moral disengagement, an athlete may minimize the importance of aggression by displacing the responsibility to the referee or by diffusing the responsibility to the aggressor’s teammates and/or coach (Boardley and Kavussanu, 2011). During a pre-match briefing in the changing room, a coach might incite the team members to be aggressive, and a referee might not see all the fouls during a match. Fourthly, willingness to forgive may be influenced by the consequences for the victim (e.g., being injured or not) (Fruchart and Rulence-Pâques, 2014). Fifthly, it would be interesting to consider the interpersonal relationship between the aggressor and victim, and the reasons for the aggressor’s actions (Maier et al., 2016). An aggressive act may enable a team to win a match and a performance bonus (Maier et al., 2016). To the best of our knowledge, the effect of this kind of incentive on forgiveness in sport has not previously been studied.

The objective of the present study was to map how amateur athletes from various sports integrated five information cues [apology, intention, moral disengagement, consequences, and incentive)] when judging the extent to which they would forgive an aggressive act in sport. We had two starting hypotheses: (i) several different positions (clusters) would be found (López-López et al., 2012; Fruchart and Rulence-Pâques, 2014; Fruchart et al., 2019), and (ii) the clusters’ respective compositions would be linked to the participants’ characteristics, i.e., the sex and the type of sporting practice (e.g., Fruchart and Rulence-Pâques, 2020).

## Methods

### Participants

We included 128 amateur athletes [92 male athletes (*M*_*age*_ = 20.25, *SD* = 1.86) and 36 female athletes (*M*_*age*_ = 21.27, *SD* = 2.64)] from various sporting backgrounds: non-contact sports (volley ball, *n* = 34), contact sports (handball, *n* = 75) and collision sports (rugby, *n* = 19). In non-contact sports, contact between opponents is prohibited and is extremely rare. In contact sports, contact between opponents is authorized under certain conditions and may be occasional or frequent. In collision sports, contact between opponents is an essential, authorized, and extremely frequent part of the activity.

All the study participants were all unpaid volunteers. We contacted potentially eligible participants at sports centers. They were given comprehensive information about the study and then asked if they wished to participate. If they agreed, we arranged an appointment. Each participant’s task was to judge their degree of forgiveness of an aggressor during various scenarios in a handball match. The scenarios were built from five factors: moral disengagement, intention, consequence, apology, and incentive.

### Material

The material consisted of a set of cards. Each card contained a brief description of the scenario, a question, and a rating scale (e.g., Anderson, 1996). The descriptions were composed according to a five within-subject factor design: Moral Disengagement (displacement of responsibility to the referee vs. diffusion of responsibility to the aggressor’s teammates or coach) × Intention (hostile aggression with intent to injure vs. instrumental aggression with intent to prevent the opponent from scoring) × Consequence (injury vs. no injury) × Apology (apology vs. no apology) × Incentive (the opposing team won the match and received a performance bonus vs. the opposing team did not win the match and did not receive a performance bonus). All possible combinations of these five factors yielded the following factorial design: 2 × 2 × 2 × 2 × 2 = 32 scenarios. The validity of these scenarios was first tested and confirmed by five amateur handball players who were very familiar with this sort of situation.

A typical scenario was as follows: “During a handball championship match, Maël is playing against Jess. There is very little time left to play, and the score is tied. Before the match, Jess’ teammates and their coach had decided to be very aggressive during the match. While defending, Jess violently pushes Maël to prevent him from scoring. Maël is injured and must leave the court. Jess apologizes to Maël. This aggressive act enabled Jess’ team to win the match, and Jess was given a performance bonus of 200 euros.”

The scenario with the opposite modalities for all five information cues was as follows: “During a handball championship match, Maël is playing against Jess. There is very little time left to play, and the score is tied. Throughout the match, the referee has failed to sanction all the aggressive behavior. While defending, Jess violently pushes Maël with the objective of injuring him. Maël is not injured and can continue to play. Jess does not apologize to Maël. This aggressive act does not enable Jess’s team to win the match, and Jess was not given a performance bonus.”

The question was “If you were Maël, to what extent would you be willing to forgive Jess?” Beneath each scenario was an 11-point response scale, ranging from “*Not at all Willing to Forgive*” on the left to “*Completely Willing to Forgive*” on the right.

### Procedure

All study procedures involving human participants were performed in accordance with the ethical standards of the local institutional review board. After obtaining the coach’s agreement, amateur athletes were tested individually before a training session. Testing took place in a quiet room in the club house. Each participant was instructed to read the scenarios (presented one at a time, in random order) describing a player who commits an aggressive act on an opponent, and to rate the degree to which they expected the aggressor to be forgiven. In line with Anderson’s (2008) procedure, our study comprised a familiarization phase and an experimental phase (see Fruchart and Rulence-Pâques, 2020).

### Data Analysis

The participants’ ratings from the experimental phase were converted into individual numerical values. In order to test our first hypothesis, a cluster analysis was performed on the whole dataset, as described previously (see Fruchart and Rulence-Pâques, 2020). A hierarchical method was performed to define the number of cluster (Ward’s method; agglomeration schedule coefficients; dendogram), then we used the *K*-means procedure to actually form the clusters (Euclidian distances). Separate repeated-measures ANOVAs were performed on the data in each cluster to identify the manner in which the members of each cluster combined factors in judgment of forgiveness. Pearson’s chi-squared test and Marascuilo’s *post hoc* chi-squared test were used to establish whether cluster groups were associated with participants’ sex and their type of practice.

## Results

### Cluster Analysis

The hierarchical cluster analysis suggested that a three-cluster solution was most likely. We performed a repeated-measures ANOVA with cluster membership as a between-subjects factor, followed by a *post hoc* test. In the three-cluster solution, the independent variable Cluster was significant, *F*(1, 125) = 5585.20, *p* < 0.001, η^2^ = 0.98. Tukey’s *post hoc* test revealed a significant difference (*p* < 0.001) between the three clusters. Hence, a three-cluster solution was selected.

### Repeated-Measure ANOVA and Graphical Analysis in Each Cluster

**FIGURE 1:**
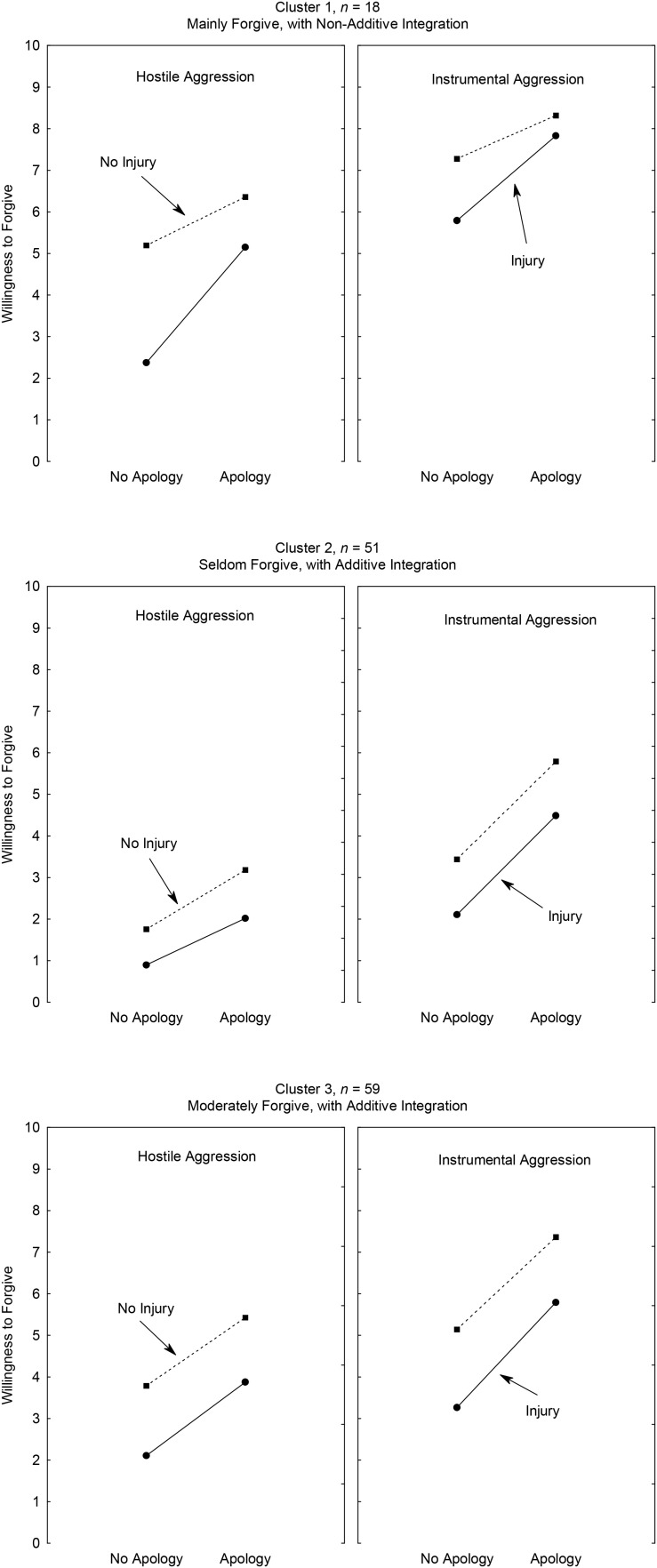
Combined effects of intention, apology and consequence factors on judgments of forgiveness for each cluster. Hostile aggression involves intent to injure the opponent; Instrumental aggression involves intent to prevent the opponent from scoring.

A repeated-measures ANOVA was then conducted on each cluster (Figures 1, 2). The results for each cluster (together with the effect sizes and margins of error) are summarized in Table 1. The mean values for each factor in each cluster are shown in Table 2. The combined effects of intention, apology and consequence factors on the judgment of forgiveness for each cluster are shown in Figure 1; each panel corresponds to a type of intention. The two levels of apology are on the x axis. Each curve corresponds to one level of the consequence factor. To name each cluster, we took account the mean response and the type of integration rule (additive or non-additive) used by the participants.

**TABLE 1:** Main results of the ANOVAs performed on cluster 1, cluster 2 and cluster 3.

	Effect		Error				
Factor	*df*	*MS*	*df*	*MS*	*F*	*p*	η^2^*_*p*_*
**Cluster 1 mainly forgive, with non-additive integration**							
Moral disengagement	1	1.562	17	2.21	0.708	0.412	0.04
Intention	1	925.17	17	14.90	62.10	0.000	0.79
Consequence	2	324.00	17	3.29	98.47	0.000	0.85
Apology	1	444.51	17	10.25	43.35	0.000	0.72
Incentive	1	0.44	17	2.31	0.19	0.666	0.01
**Cluster 2 seldom forgive, with additive integration**							
Moral disengagement	1	21.66	50	5.66	3.82	0.056	0.07
Intention	1	1616.04	50	8.34	193.79	0.000	0.80
Consequence	1	553.00	50	6.01	92.01	0.000	0.65
Apology	1	1353.06	50	4.64	291.21	0.000	0.85
Incentive	1	128.53	50	2.66	48.38	0.000	0.49
**Cluster 3 moderately forgive, with additive integration**							
Moral disengagement	1	34.71	58	11.02	3.15	0.081	0.05
Intention	1	1194.92	58	9.74	122.71	0.000	0.68
Consequence	1	1315.56	58	7.35	179.06	0.000	0.76
Apology	1	1968.85	58	6.45	305.36	0.000	0.84
Incentive	1	48.30	58	4.24	11.39	0.001	0.16

**TABLE 2:** Mean and *SD* scores for each factor in the three clusters.

	Cluster 1 mainly forgive, with non-additive integration		Cluster 2 seldom forgive, with additive integration		Cluster 3 moderately forgive, with additive integration	
	*M*	*SD*	*M*	*SD*	*M*	*SD*
	6.04	0.18	2.96	0.09	4.59	0.06
**Factor moral disengagement**						
Displacement of responsibility on to the referee	6.09	0.20	3.07	0.10	4.73	0.09
Diffusion of responsibility on to the teammate and coach	5.98	0.17	2.84	0.11	4.46	0.10
**Intention**						
Hostile aggression with intention to hurt	4.77	0.28	1.96	0.10	3.80	0.11
Instrumental aggression with intention to prevent the opponent from scoring	7.31	0.19	3.95	0.13	5.39	0.07
**Consequence**						
Injury	5.28	0.23	2.38	0.10	3.76	0.85
No injury	6.79	0.15	3.54	0.11	5.43	0.09
**Apology**						
No apology	5.16	0.25	2.05	0.08	3.57	0.08
Apology	6.91	0.19	3.87	0.12	5.61	0.09
**Incentive**						
The match has not been won and a match performance bonus has not been given	6.01	0.19	3.24	0.10	4.75	0.07
The match has been won and a match performance bonus has been given	6.01	0.19	2.68	0.09	4.43	0.08

Cluster 1 (*n* = 18) was named “Mainly Forgive, with Non-Additive Integration” because the mean response (*M* = 6.04; *SD* = 0.18) was above the midpoint on the 0–10 scale, and the curves were not parallel (i.e., they indicated a non-additive integration rule; top panels in Figure 1). The members of this cluster judged that the victim of the aggression would be willing to forgive the aggressor in most scenarios. The curves rise, which indicates the effect of apology. The curves are separate, which indicates an effect of consequence. The curves in the right panel are higher than the ones in the left panel, showing an effect of intention. In both panels, the curves converge and rise to the right; hence, the members of this cluster applied a non-additive rule. The Apology × Consequence interaction was significant, *F*(1, 17) = 34.54, *p* < 0.001, η^2^*_*p*_* = 0.67. The Intention × Apology × Consequence interaction was not significant, *F*(1, 17) = 3.36, *p* < 0.234, η^2^*_*p*_* = 0.08.

Cluster 2 (*n* = 51) was named “Seldom Forgive, with Additive Integration,” since the mean response (*M* = 2.95, *SD* = 0.89) was close to the low (left) side of the 0–10 scale (middle panels, Figure 1) and since the curves were parallel (additive rule). The members of this cluster judged that the victim of the aggression would rarely be willing to forgive the aggressor. The curves rise (showing the effect of apology) and are separate (showing the effect of consequence). The curves in the right panel are higher than ones in the left panel, demonstrate the effect of intention. The Apology × Consequence interaction was not significant, *F*(1, 50) = 0.85, *p* < 0.361, η^2^*_*p*_* = 0.02. The Intention × Apology × Consequence interaction was not significant, *F*(1, 50) = 1.79, *p* < 0.187, η^2^*_*p*_* = 0.03.

Cluster 3 (*n* = 59) was named “Moderately Forgive, with Additive Integration,” since the mean response (*M* = 4.59; *SD* = 0.06) was near the midpoint of the 0–10 scale and the curves were parallel (bottom panels, Figure 1). The members of this cluster judged that the victim of the aggression would be moderately willing to forgive the aggressor. The curves are separate (indicating an effect of consequence), have a clear slope (indicating an effect of apology), and are parallel (showing that the integration rule was additive). The curves in the left panel are lower than ones in the right panel, which indicates an effect of intention. The Apology x Consequence interaction was not significant, *F*(1, 17) = 1.46, *p* < 0.232, η^2^*_*p*_* = 0.02. The Intention x Apology x Consequence interaction was not significant, *F*(1, 58) = 0.326, *p* < 0.570, η^2^*_*p*_* = 0.01.

Table 3 shows the composition of each cluster with regard to the participants’ sex. The 2 (male/female) × 3 (clusters) Pearson’s chi-squared test was significant, χ^2^ (2) = 22.549, *p* < 0.001. The overall chi-squared test showed that there were some intergroup differences but that not all subgroups differed from others. Hence, Marascuilo *post hoc* chi-tests were conducted. The 2 (sex) × 2 (cluster 1-cluster 2) Marascuilo chi-squared test was significant, χ^2^(1) = 11.53, *p* < 0.001. Nineteen percent of the women were in the first cluster, and 72% of the men were in the second cluster. The 2 (sex) × 2 (cluster 2-cluster 3) Marascuilo chi-square test was significant, χ^2^(1) = 16.09, *p* < 0.001; 54% of the women were in the third cluster, and 72% of the men were in the second cluster. The other 2 (sex) × 2 (clusters) Marascuilo chi-square test were not significant (*p* > 0.05).

**TABLE 3:** Composition of the clusters, with regard to the participants’ type of sport and sex.

	Cluster 1 mainly forgive, with non-additive integration	Cluster 2 seldom forgive, with additive integration	Cluster 3 moderately forgive, with additive integration	Total
**Type of sport**				
Non-contact	2 (6%)	18 **(53%)**	14 (41%)	34
Contact	10 (13%)	31 **(43%)**	34 (45%)	75
Collision	6 **(32%)**	2 (10%)	**11 (58%)**	19
Total	18	51	59	128
**Sex**				
Female	17 **(19%)**	25 (27%)	50 **(54%)**	92
Male	1 (3%)	26 **(72%)**	9 (25%)	36
Total	18	51	59	128

*Bold values indicate *p* < 0.05.*

Table 3 shows the composition of each cluster in terms of type of practice of participants too. The 3 (non-contact sport, contact sport, and collision sport) × 3 (Clusters) Pearson’s chi-square test was significant, χ^2^(2) = 8.443, *p* < 0.016. The 3 (type of sport) × 2 (cluster 1-cluster 2) Marascuilo chi-square test was significant, χ^2^(2) = 12.67, *p* < 0.001. The first cluster contained 32% of the participants from collision sports, and the second cluster contained 53% of the participants from non-contact sport and 43% of the participants from contact sports. The 3 (type of sport) × 2 (cluster 1-cluster 3) Marascuilo chi-square test was significant, χ^2^(2) = 6.32, *p* < 0.04. The third cluster contained 58% of the participants from collision sports, and the first cluster contained 32% of the participants from collision sport. The other 3 (type of sport) × 2 (clusters) Marascuilo chi-square test were not significant (*p* > 0.05).

## Discussion

The objective of our study was to map the way amateur athletes from various types of sport combined five information cues when judging whether to forgive an aggressor in a sporting situation. The first hypothesis [based on literature data from the application of information integration theory to sport (e.g., Fruchart and Rulence-Pâques, 2020) and judgments of forgiveness in politic (López-López et al., 2012)] was that different views of forgiveness would be found. This hypothesis was confirmed by the presence of three different clusters. The second hypothesis was that the clusters’ composition would be related to the members’ characteristics. This was also confirmed by the study results; the clusters’ composition was linked to the type of sport and to the sex.

The members of the first cluster (i) judged that the aggressor would be usually be forgiven, and (ii) applied a non-additive integration rule. The cluster contained 19% of the female participants but only 3% of the male participants. It contained 32% of the athletes from collision sports but only 13% of the athletes from contact sports and 6% of the athletes from non-contact sports.

The members of the second cluster (i) judged that the aggressor would seldom be forgiven, and (ii) used an additive integration rule. The cluster contained 72% of the male participants and 27% of the female participants. It contained 53% of the athletes from non-contact sports, 43% of the athletes from contact sports and only 10% of the athletes from collision sports.

Lastly, the members of the third cluster (i) judged that the aggressor would moderately be forgiven, and (ii) used an additive rule. The cluster contained 54% of the female participants and 25% of the male participants. It contained 58% of the athletes from collision sports, 45% of the athletes from contact sports, and 41% of the sports from non-contact sports.

A similitude between the 3 clusters were found. The Moral disengagement factor was not significant in each cluster. The participants did not differentiate the displacement of responsibility to the referee and the diffusion of responsibility to the aggressor’s teammates or coach in judgment of forgiveness. We can explain this finding by relating it to the identical goal of these types of moral disengagement which is to minimize the importance of aggression (Boardley and Kavussanu, 2011).

Our findings confirm that there are inter-individual differences in the integration rules used to judge forgiveness in sport, as in politics (López-López et al., 2012) and to make judgments in sport (e.g., Fruchart et al., 2019). These differences can be revealed by cluster analyses (e.g., Fruchart and Rulence-Pâques, 2020) and the application of information integration theory (Anderson, 2008). These approaches enabled us to clearly map different positions with regard to cognitive processes in sport-related judgments.

Our results also confirmed the link between the individuals’ characteristics and their position with regard to judgment in sport (Fruchart and Rulence-Pâques, 2020). Just as the type of involvement in sport is associated with cluster composition in ethical judgments (Fruchart et al., 2019), the type of sport practiced was linked to the cluster composition in judgments of forgiveness. The participants from non-contact sports or contact sports judged that they would less forgive the aggressor. In contrast, the participants from collision sports estimated that they would more forgive the aggressor. Being confronted with high degree of contact might prompt sportspeople to forgive their aggressor more easily. This suggests that aggression is acceptable in high-contact sport and is viewed as a consequence of a high level of physical contact. The participants would therefore be willing to forgive the aggressor in certain situations. This finding may be explained by the social perspective of aggression discussed by Gabler (1976), which highlights the difference between prohibited aggressive acts and thus that are allowed, depending on the norms of the game. This attitude to aggression in sport showed that athletes have very firm beliefs about what is “aggressive” or “not aggressive,” depending on their sport (Bar-Eli et al., 2016).

The participant’s sex was also linked to with the clusters’ composition. The male participants tended to forgive the aggressor quite rarely, whereas the female participants would consider forgiving the aggressor occasionally. That is coherent with psychology research in which (i) females were more likely to forgive than males, and (ii) the relationship between sex and forgiveness was explained by empathy (e.g., Toussaint and Webb, 2005). However, our result conflicts with other studies of the effect of sex on forgiveness in sport (Gauché and Mullet, 2005).

Our study had a number of limitations. Firstly, one limitation was the choice of sport type for the scenarios. Handball was the sport for which the scenarios were described and some of the amateur athletes played this sport, whereas others did not. This will likely also influence the participants’ willingness to forgive the aggressor since handball players are probably more familiar with the described scenarios and might take other information cues into account than non-contact or collision sport athletes that are confronted with a scenario in a sport (handball) that they do not practice. This present study rather highlighted how athletes of different sport types judge situations within a contact sport and what degree of aggression in this contact sport appears to be appropriate from their view. Secondly, the participants were amateur athletes; by analogy with studies of ethical judgment in sport, it would be interesting to evaluate forgiveness in professional athletes as well (Fruchart and Rulence-Pâques, 2014). Thirdly, in contrast to Gauché and Mullet (2005), we did not study the impact of psychological aggression on the willingness to forgive. Fourthly, we did not use a personality questionnaire [such as the Dispositional Forgiveness Questionnaire (Mullet et al., 2003)] to gain additional information on each individual’s willingness to forgive and to test the effect of this willingness on the judgment on forgiveness. Fifthly, the sample were not balanced between men and women or by the type of sport they practice.

One scientific finding from this study is that the identification of integration rules can reveal different positions with regard to forgiveness in sport. This kind of study could be applied to human relationships in sport. Various relationships between the sports actors (athletes, coaches, parents, medical staff, sports psychologist…) may be explored in considering the influence of social psychological process in performance (Carr, 2012). Exploring the judgment of forgiveness in these relationships could be useful for stakeholders involved in sport.

## Data Availability Statement

The raw data supporting the conclusions of this article will be made available by the authors, without undue reservation.

## Ethics Statement

Ethical approval was not provided for this study on human participants because our institution does not provide ethical approval. The patients/participants provided their written informed consent to participate in this study.

## Author Contributions

Both authors participated in the methodology and the redaction of the article. Both authors contributed to the article and approved the submitted version.

## Conflict of Interest

The authors declare that the research was conducted in the absence of any commercial or financial relationships that could be construed as a potential conflict of interest.
